# Exploring perceived barriers to palliative and end of life care provision in South-West England: bringing together the perspectives of professionals, patients, and families

**DOI:** 10.3389/fsoc.2024.1488688

**Published:** 2025-01-20

**Authors:** Gary Hodge, Gina Kallis, Tomasina M. Oh, Hannah Wheat, Susie Pearce

**Affiliations:** ^1^School of Nursing and Midwifery, University of Plymouth, Plymouth, United Kingdom; ^2^Peninsula Medical School, University of Plymouth, Plymouth, United Kingdom; ^3^Torbay and South Devon NHS Foundation Trust, Torbay, United Kingdom

**Keywords:** palliative care, patient, professionals, family carers, hospice care, end of life care

## Abstract

**Introduction:**

Palliative and End of Life care (PEoLC) in the United Kingdom (UK) is increasingly being reported as inadequate. This is occurring amidst a wider backdrop of health and social care systems facing unprecedented pressure, particularly as they recover from the long-term impact of the COVID-19 pandemic. This study aimed to explore the barriers to PEoLC faced by those providing and receiving care in South-West England (UK). This region of the UK brings its own set of unique challenges due to its rural and coastal location, an aging population, and a historical lack of research.

**Methods:**

An exploratory study was conducted which involved patients, families, and professionals who were providing and receiving PEoLC. A total of 13 qualitative focus groups were held with a total of 63 participants; 45 were health and care professionals and 18 were people toward the end of their life, family/carers and people who were bereaved.

**Results:**

A range of barriers were identified for those providing and receiving PEoLC services. These were a lack of specialist palliative and EoL care resources (particularly in out-of-hours care); poor communication, collaboration and co-ordination across providers; inequalities in the access and provision of care; the impact of the COVID-19 pandemic; and a reluctance to have conversations about death and dying.

**Conclusion:**

This study brings together the voices of patients, family, and professionals from different settings in a geographical area of the UK. Understanding their experiences and perceived barriers to care is key to being able to develop and transform care. Ultimately, there is a need for a collaborative and co-ordinated approach across both practice and research, working toward what is important to those providing, and most importantly, those receiving care at the end of their lives.

## Introduction

Palliative and end of life care (PEoLC) care is an essential aspect of health and care provision that addresses the complex needs of patients with life shortening conditions, as well as their families and caregivers. Its focus is to improve quality of life and relieve suffering for both patients and their carers (Sleeman et al., 2021). In England, the end of life aspect of PEoLC refers to a person's last 12 months of life (NHS England, 2022). Despite advancements in medical treatments and supportive care, the delivery of PEoLC continues to face numerous challenges, particularly in resource allocation, communication, care co-ordination, and inequitable access to care [Health Services Safety Investigations Body (HSSIB), 2023; NHS England, 2021].

The demand for palliative care in the UK is increasing due to the aging population and the rising prevalence of multiple long-term conditions (UK Parliament, 2022). However, there is a documented shortfall in specialist palliative care services, both in hospital and community settings (Mason et al., 2022). In addition to these well documented demographic projections and the limited resources provided to PEoLC services, the COVID-19 pandemic also had a lasting impact. The All-Party Parliamentary Group [The All-Party Parliamentary Group (APPG) on Hospice and End of Life Care, 2023] on hospice and end of life care found that the sheer volume of death brought about by the pandemic not only highlighted significant gaps in care, but also resulted in exhausted health and care workers and unpaid carers. It was, however, noted that lessons can be learnt from the COVID-19 pandemic, for example the continuation of innovative and collaborative working shown during the pandemic; although this needs to be properly funded, commissioned and sustained (Marie Curie, 2024). How and where PEoLC funding is allocated is a global issue which requires rebalance. Many low-mid income countries are dependent on charity, in contrast to high-income countries which have focused significant annual health expenditure on the medicalization of death and dying (Sallnow et al., 2022). Although the COVID-19 pandemic negatively impacted on many communities around the world, it was particularly challenging for groups who already faced health, socio-economic and access to care inequalities (British Medical Association (BMA), 2022). A range of factors play a role in the inequalities of access to care. These include disease group, socio-economic status, rurality, and ethnicity to name just a few (Baylis et al., 2023).

Despite the inequalities often experienced by people near the end of life, they are underrepresented in health and medical research (Johnson et al., 2021). In the UK, there is only a small body of work which explores their experiences and perspectives (Almack et al., 2012; Conner et al., 2008; Costello, 2001; Janssen and MacLeod, 2010; Law, 2009; Mason et al., 2022; Mayland et al., 2021; Payne et al., 2010; Reeve et al., 2012; Worth et al., 2006). Research has examined the need for person-centered care near the end of life (Connolly et al., 2016; Sampson et al., 2020) and has drawn attention to the complexities surrounding communication between patients and health and care professionals in planning for PEoLC (Almack et al., 2012; Reeve et al., 2012). Studies have shown a need for clearer information on how to access out-of-hours health and care services (Mason et al., 2022) and for these services to consider the complex needs associated with PEoLC (Worth et al., 2006). Recent research has found the need for improved out-of-hours care at home (Pask et al., 2022) especially in regions where there are also inequalities in access to and quality of PEoLC services (National Palliative and End of Life Care Partnership, 2021).

The study presented in this paper was based in South-West England. This is a region that presents its own unique set of challenges. In 2018, the South-West Peninsula (Devon, Cornwall, and Somerset) had the highest percentage of population aged 65 and above compared to other areas in England (Hansford et al., 2023). The area is largely rural with an extensive coastline. Research has found that those living in rural areas face additional challenges accessing health and care services (Hospice UK, 2021).

The study presents the findings from 13 focus groups that involved a wide range of participants involved in PEoLC across this geographical area. Participants included health and care professionals, people who were toward the end of their life, family members who were providing care, and people who were bereaved. This study aimed to explore the barriers to PEoLC provision faced by those providing and receiving care in South-West England (UK). A secondary aim of the study was to identify priorities for future research. This is not covered in this paper as this part of the study has been developed into a follow-up research priority setting survey. This paper portrays the multifaceted nature of PEoLC by capturing what is perceived as important to participants who provide and receive PEoLC and the barriers to care they face, by drawing on their professional and personal experiences and insights.

## Methods

The study utilized an interpretative exploratory qualitative methodology which incorporated the perspectives of patients, families, and health and care professionals. Interpretive methodology is an approach which can be used when a social reality of a particular group or community needs to be explored and understood through a process of sensemaking (Given, 2008). Focus groups were selected as the qualitative method to collect data on participants' stories because they facilitate discussion and exploration of experiences and perceptions with groups of people who have had similar experiences (Kitzinger, 2013). This was considered helpful to explore more deeply the issues, challenges and priorities for care around the end of life for different groups. Focus groups were held separately with health and care professionals, people toward the end of life, their family members/carers, and people who were bereaved. In some cases, due to the nature of the topic only one or two people in the patient/family focus groups attended (Table 1). When this happened, an interview was facilitated rather than a focus group. All focus groups and interviews were conversational in style and were guided by the participant(s) rather than the facilitator. This allowed the conversation to flow and individual's experiences to be captured.

**Table 1:** Focus group and interview participants.

**Focus group/interview no**.	**Number of participants**	**Professional/patient/family**
1	5	•Hospital nurse consultant •Community nurse consultant •Community team manager •Education team member (hospice) •Clinical nurse specialist
2	7	•2 × in-patient unit nurses (hospice) •2 × senior managers (hospice) •Health care assistant (hospice) •Senior nursing support worker •Governance team member (hospice)
3	5	•Social worker •Student nurse •Health care assistant (hospice) •Director of patient care (hospice) •Referral co-ordinator
4	8	•2 × education team members (hospice) •Education team/in-patient unit nurse (hospice) •Lead palliative and end of life care specialist nurse (hospital) •Occupational therapist •Advanced nurse practitioner (hospice) •Student nurse •Community hospital nurse
5	7	•Medical consultant and research lead (hospice) •Adult/specialist nurse •Community team leader •Hospital palliative care nurse •Community palliative care nurse •Junior nursing sister on in-patient unit (hospice) •Research nurse (hospice)
6	6	•2 × in-patient unit nurses (hospice) •2 × community team leaders (hospice) •Occupational therapist (rehabilitation team) •Team leader on in-patient unit (hospice)
7	7	•2 × district nurses •Bereavement support volunteer •Palliative and end of life care leader •General practitioner •Occupational therapist •Community palliative care clinical nurse specialist
8	2	•Patient [diagnosed with motor neurone disease (MND) diagnosed 2022] •Family carer (wife) of patient above
9	5	•Bereaved husband (wife died in 2022) •2 × bereaved parents—(daughter died in 2022) •Bereaved husband (wife died in 2022) •Bereaved wife (husband died in 2022)
10	1	•Bereaved wife (wife died several years ago)
11	2	•Bereaved partner (partner died in 2016) •Bereaved husband (wife died in 2022)
12	1	•Patient (secondary breast cancer diagnosed 2008)
13	7	•Patient (palliative care commenced 2023—diagnosis not shared) •Patient (diagnosed with cancer 2019) •Patient (diagnosed with cancer 2022) •Family carer (partner) of patient above •Family carer (wife) (husband diagnosed with kidney cancer 2022) •Patient (diagnosed with pancreatic cancer 2021) •Patient (diagnosed with cancer in 2022).

### Participant recruitment

Potential participants were invited to take part by six hospices in South-West England. Study information was sent out via email to their professional and patient/family networks. Professional networks spanned across specialist palliative care, community teams, acute and community hospitals. Those who expressed an interest in taking part in the study were contacted by a member of the research team or a key contact from the hospice. Thirteen focus groups were held with a total of 63 participants- 45 who were health and care professionals and 18 who were patients, family members, and carers. The numbers of participants attending each group are displayed in Table 1. Focus groups took place in June/July 2023.

### Data collection

This paper focuses on one of the study aims of exploring the barriers to PEoLC provision faced by those providing and receiving care in South-West England (UK). To do this, data was collected through focus groups and interviews. All focus groups and interviews were held in person, except for one professional group, which was hosted virtually. In-person focus groups and interviews took place on hospice sites, apart from one interview with two people which was held at a local community center. This was because it was more central and easier to get to than the hospice site for the participants. All sessions were facilitated by an experienced qualitative and PEoLC researcher with a nursing background and experience of conducting focus groups (SP) and co-facilitated by one other experienced qualitative researcher (GH, GK, HW). All sessions lasted between 1 and 2 h, 25 min.

All focus groups and interviews were audio recorded, and field notes were captured immediately afterwards. During the focus groups/interviews, professionals were asked about their experiences of working with people at the end of life, what they believed was important in the delivery of care, and the challenges and barriers to care provision they faced. Patients and family members were asked about their experiences in relation to either themselves or someone they cared for. They were also asked what they believed was important in the care they or their family member had received, and what barriers to care they had experienced. As the focus groups and interviews were conversational in style, a formal and structured set of questions were not set and instead, as previously highlighted, the conversational flow of the focus group or interview was guided by the participants, prompted only by the questions shared above.

The focus groups were for some of the patient and family participants the first time they had talked about some of their experiences of what had happened or was happening to them. The health and care professionals also found themselves together in groups discussing and sharing some difficult issues in ways they do not often have the chance to do. All the focus groups were facilitated to provide a shared and supportive space. There was time to connect and chat before the groups, and the researchers stayed in the room for some time afterwards to allow the opportunity for further conversation and debriefing. Focus group venues were chosen for patient and family focus groups so there was a quiet space to go if a break was needed.

In all the patient and family focus groups/interviews there was a hospice member of staff nearby or contactable if further follow-up or support was needed. The participants all appeared to find the opportunity to talk and tell their stories beneficial. One participant was not well and had problems accessing primary care services and so they were connected with hospice staff straight after the focus group. Researcher reflection, reflexivity, and debriefing (Karcher et al., 2024) was also essential to this study. The researchers met to debrief after the focus groups and reflexive field notes were maintained.

### Data analysis

All recordings were professionally transcribed verbatim and anonymized. Transcripts were then uploaded to NVivo 12 software to help organize the data. An iterative approach to analysis was undertaken that followed the principles of reflexive thematic analysis (Braun and Clarke, 2019, 2022). Codes were generated inductively from the data. To enhance coding rigor (Finlay, 2021), a team of five researchers (GH, GK, TO, HW, SP) independently analyzed one/two transcripts each, generating and sharing a selection of codes. Three of the researchers then reviewed all these codes (GH, GK, SP). Following discussion, the codes were integrated into themes. These were reviewed, refined, and developed to generate five key themes: a lack of palliative and end of life care resources; poor communication, collaboration, and co-ordination across providers; inequalities in the access and provision of care; the impact of the COVID-19 pandemic; and a reluctance to have conversations about death and dying. Reflexive discussions and de-briefing took place with the research team during data analysis. This allowed the sharing of thoughts, uncovered potential biases, promoted transparency, and reduced subjectivity (Ahmed, 2024).

### Ethical considerations

Ethical approval was obtained from the University of Plymouth's Faculty Research Ethics and Integrity Committee (FREIC). All participants received a Participant Information Sheet and provided written informed consent before participating. The research team worked closely with a member of each hospice to ensure all participants were supported during focus groups, and further support was available and offered in case of distress.

## Results

### Demographic characteristics

Seven focus groups were held with 45 professionals: Nurses (*n* = 12); Manager/Leadership Roles (*n* = 10) Specialist/Advanced Nurse Practitioners (*n* = 7); Health Care Assistants (*n* = 3); Occupational Therapists (*n* = 3); Hospice Educational Roles (*n* = 3); Medics (*n* = 2); Student Nurses (*n* = 2); Administrative Roles (Quality/Governance (*n* = 2) and a Bereavement Support Volunteer (*n* = 1). Another six focus groups were held with 18 people toward the end of life/being supported by PEoLC (Patients *n* = 7), family members/carers (*n* = 3) and people who were bereaved (*n* = 8). Not all patient participants shared their diagnosis, which was respected by the researchers. However, of those who did the majority had received a cancer diagnosis (*n* = 5). Details of participants attending each focus group/interview are displayed in Table 1.

## Themes

The section below presents the findings across five key themes, utilizing participant quotes. A range of barriers to PEoLC provision were identified across services. These were a lack of specialist palliative and EoL care resources (particularly in out-of-hours care); poor communication, collaboration and co-ordination across providers; inequalities in the access and provision of care; the impact of the COVID-19 pandemic; and a reluctance to have conversations about death and dying.

### A lack of palliative and end of life care resources

One of the most frequently discussed barriers to care for people toward the end of life highlighted by participants was the lack of resources. Professionals who participated discussed the lack of specialist resources in PEoLC services and the difficulties organizations experienced in identifying and recruiting staff with the relevant specialist skills:

“*I think workforce wise, just having the number of staff to deliver the care. Much like any other healthcare setting. But also finding staff who either have that specialist skill to be able to deliver palliative care or to be able to train them in that specialist skill…we are having to step up and do things quite routinely that we wouldn't have done before.”* (Professional, Focus Group 2)

Linked to this, hospice staff felt there was a lack of understanding among the public and health and care providers more generally around the role of hospices and the fact that the care they provide is specialist:

“*There's a lot that goes on and an awful lot around actively making sure somebody feels as well as they can. And that does sometimes become very medical, which it needs to. And I don't think that's always fully understood either that it's an active specialism…some people still think we're just a nursing home up here. It is extraordinary really.”* (Professional, Focus Group 3)

Some participants felt that this lack of understanding resulted in a lack of funding allocation, as well as a lack of referrals for certain types of care. The need for more specialist provision also extended to home-based and out-of-hours care:

“*Care at home is provided by agencies who provide generalized care at home for all sorts of different patients. So, it's not specialist. Yes, they will have had some end of life care training but it's not specialist by any stretch.”* (Professional, Focus Group 2)

More generally, professionals raised concerns about how the lack of out-of-hours care provision impacted on patients and families. For example, there were accounts of both professionals and family members struggling to access out-of-hours medication. This struggle was exacerbated in rural locations where individuals often had to travel longer distances between pharmacies:

“*Out-of-hours pharmacies on weekends… they just don't have stock. And you're sending the family off to collect some meds. Don't have them. There's no communication with the family that we don't. So, they drive there. They've left their, their loved one on their own just for a bit. Or got a neighbor to come and sit with them…I think it's the lack of ownership sometimes, isn't it, with people that work in chemists.”* (Professional, Focus Group 5)

As well as issues accessing medication, professionals were aware that patients and families often needed support when at home or during evenings and weekends, but were not able to get through to relevant organizations/service providers:

“*Lack of available services out there for them [patients and families]. When they're having that moment of crisis in the middle of the night, and they can't get anybody to respond to their calls.”* (Professional, Focus Group 2)

“*We've got an advice line service overnight that the ward provides…we do have quite a few frantic carers or – calling in, all saying, “We've called and nobody, nobody's getting back to us.” They're in like an acute crisis at that time, and they can't get people to them. I think there definitely – that's definitely an issue at the minute.”* (Professional, Focus Group 2)

In line with the above accounts, bereaved family members recounted instances where they had struggled to access home-based support when caring for loved ones and sometimes resorted to calling emergency services or turning up at an emergency department. This was not necessarily appropriate based on their needs at the time, and the professionals they saw were often unfamiliar with the patient's medical history. Several participants believed that having a single point of contact in times of need would have been beneficial:

“*I think that if, if it was possible – which I think it should be possible – but I've had this conversation so many times over the decades, you know, as a GP, as a carer – to have where families can have a single point of contact that they know, if they ring that number, something will happen. Somebody will take the pressure off them. That they don't have to keep making that same phone call and going through the same information to different people who don't know you, don't know the person you're looking after in this case. But in general it surely can't be impossible.”* (Bereaved husband, Focus Group 9)

Following the death of a loved one, some family members felt there was a need for more bereavement support. There were some instances where families had been offered bereavement support immediately after the death, which they felt was too soon. There were others who had not been offered any support and/or did not know how to access it.

Some professionals also noted how the lack of follow-up with people who were bereaved could leave them feeling discarded:

‘*”cause of staffing we're losing those, those face-to-face sort of in-person bereavement sort of visits with families which can really help wrap things up and show compassion and empathy and all the rest of it.”* (Professional, Focus Group 7)

### Poor communication, collaboration, and co-ordination across providers

As well as a lack of resources, issues were identified around communication, collaboration and co-ordination across providers. Participants felt that specialist PEoLC services needed to effectively share information between them, which was up-to-date and accurate. Some participants reported that this was not always the case:

“*…giving information, they've come into the hospice on that information, and we then turn around and say, “Actually, that's not correct.” And then they're not very happy that they have a relative, or they are the patient on the ward, and it's not the correct information that they've been given. So, it can be really difficult.”* (Professional, Focus Group 2)

Inaccurate or omissions in information between providers was found to be particularly challenging for the receiving service (Hospice). This meant that there were times when patients and their families believed they were still on active treatment pathways, as they had not been told that they had moved into a palliative treatment phase:

“*…families not being informed...then that is a challenge for practitioners who can see that someone's perhaps dying but they're still on the sort of trajectory for active treatment…It just hasn't been communicated to them that maybe they're having palliative treatment, and they think they're having active treatment still.”* (Professional, Focus Group 1)

Errors concerning the sharing of information not only impacted on the professionals working across health and care services, but also the patients. One patient shared how distressing it was when information around their treatment was omitted or ignored:

“*I've been asked all sorts of questions… “so, when are you having your operation?” “Well, actually, I'm not having one.” And they shouldn't really be asking those sort of questions. You know, it's very distressing to, to ask me a question when I've been told I haven't got that long, and I know I can't be operated… They shouldn't ask if they don't know the answer. And things like that have been very, very poor.”* (Patient, Focus Group 13)

Professionals believed that gaps in information or understanding were exacerbated by a lack of collaboration across care networks and pathways. One professional suggested that this lack of collaboration not only negatively impacted on communication, but also led to delays in process:

“*What has been apparent over the years that I've been privy to being with palliative and end of life care – over 30 years now – is the fact that we're not a completely collaborative service and the fact that we are unfortunately under different umbrellas. And that can make processes very, very prolonged and delayed, especially around communication…I think to have a much more cohesive, collaborative approach under the one umbrella would have many, many benefits...”* (Professional, Focus Group 4)

When services were able to work more collaboratively, professionals reported that information shared was used more effectively, and this had a positive impact on relationships between and across multi-disciplinary teams:

“*What I see as a positive is the fact that as a hospice – we do work closely with a clinical nurse specialist – the communication between, I think, their teams and us is actually quite good. We can pick the phone up to them at any given time. And if – we can to the consultant... So, I think that's a real positive, you know. We've got very much that sort of relationship. And it's very rare that doesn't work.”* (Professional, Focus Group 6)

Participants reported that the lack of co-ordination and continuity across providers was especially challenging for patients and family carers. The professional below conveyed the challenges those who decided to die at home faced navigating between services and how confusing and frightening that navigation could be:

“*…one of the main issues we're having...in my area is continuity for the patient when someone decides they want to die at home, there are so many different services involved in caring for that person and there is no single one agency whose kind of overseeing that care. And the patient and the family don't know who to phone up for what. And they're very confused and it's a scary time…And it's very difficult for patients to navigate and you know, to get the right care at the right time.”* (Professional, Focus Group 7)

The confusion of who patients and families were supposed to contact when they had a question or issue was highlighted by both patients and family members:

“*I've got at home – I've got a whole list of phone numbers. You know, people have – just keep giving me. I've got a district nurse, community nurse, hospice nurse. Other people, the doctor. And as I say, you don't know who to contact with a specific query… sort of thing.”* (Family carer, Focus Group 13)

### Inequalities in the access and provision of care

Various inequalities in access and PEoLC provision were discussed by the participants in this study. These inequalities were linked to rurality, the challenges of providing care to diverse groups and whether patients were receiving hospice care or not. Professionals who were based in rural locations discussed some of the specific challenges they experienced working in large rural counties. They observed that due to their remoteness, rural areas had less services and resources than more central towns and cities, and this affected the care that patients received:

“*It's almost a postcode lottery. I don't like to use that term, but it is, isn't it. Because we're obviously quite remote here. Whereas cities obviously have more.”* (Professional, Focus Group 6)

In particular, this remoteness affected the provision of out-of-hours care and professionals discussed how services were either not in place, or it was difficult to find staff to provide care:

“*Especially in a county like [this]. You know… it's a nightmare… If you're in the central part of [the county] you might be lucky to get something but if it's bank holiday and weekend forget it, if you live down west. You know, when you've only got two buses a week or whatever and the school holidays and most of the care staff that are going to provide the care have children so you're not going to have that opportunity. So, it's never changed.”* (Professional, Focus Group 4)

The impact of poor transport links in rural locations was a concern raised by several participants. Professionals were aware that patients often had to travel long distances to get to appointments. This was made even more challenging when they struggled to find the appropriate transport to do so:

“*One of the key things from the community point of view is actually access and transport. That's been a huge issue for quite a number of our patients in the community in terms of getting to (appointments) ‘cause very often they have to go down to (cities) or even into (town) which is a bit of a trek. And actually, finding affordable transport and reliable transport's a real challenge.”* (Professional, Focus Group 6)

Similarly, professionals shared how their response times were often longer for those living in remote areas, as it took longer for them to travel to patients. They expressed that this could be distressing when patients were in pain and waiting for symptom control. Some patients and family members also described how far they had to travel to get to some appointments, especially when they were under the care of larger hospitals based in the nearest city, which could be many miles away:

“*We would have had to gone a long way for the […] face-to-face ‘cause […] while he (husband) was under (hospital care) with his kidney thing and his – the consultant was right down at (area). You know, it's a long way for us to go sort of thing. 50 miles.”* (Family carer, Focus Group 13)

As well as the inequalities caused by rurality, professionals were aware of the difficulties of providing accessible and inclusive care to all groups of people, particularly disadvantaged and seldom heard groups. Several participants discussed how their organizations could potentially be more inclusive, as one professional stated:

“*One thing is to recognize some people don't even get the access to be able to recognize. And so, I was thinking about, you know, different ethnic minorities or about making sure we're inclusive within homelessness, within the prison, with people that just don't sit with the support around them that they need to have.”* (Professional, Focus Group 2)

Most of the hospices were working with other organizations to consider how they could broaden their reach; however, participants were aware there was more work to be done in this area. There were also some professionals who worried that, despite the focus on inclusivity, there were still individuals who were slipping through the net:

“*It's funny, isn't it, I know disadvantaged groups are a focus, but I worry about people who fall just in between all of these specialist areas.”* (Professional, Focus Group 3)

Similarly, a couple professionals discussed how patients received varying levels of care depending on whether they were under hospice care or not:

“*I just also wonder about the difference between patients who do see the hospice…a lot of my patients who are not under hospice care but who are dying, who have long-term conditions like dementia, were actually… the care is very, very different and we rely heavily on our wonderful community nurses… And I still think there is a two-tier service.”* (Professional, Focus Group 7)

### The impact of the COVID-19 pandemic

It is widely documented how managing PEoLC became extremely challenging across settings during the peak of the COVID-19 pandemic (Glass et al., 2020; Spacey et al., 2021). Although focus groups took place sometime after this, the impact of the pandemic was still being felt by participants. In terms of practical impacts, professionals described how the pandemic exacerbated some of the issues which resulted in delayed diagnosis of patients. Subsequently, patients were referred to hospices and palliative care far later, meaning they provided care to them for much shorter periods of time:

“*We did go through a run with Covid, didn't we? With hospice at home, it was about 48 hours we had patients (before dying). That's not appropriate. And some of that is delayed diagnosis and all of those other factors and problems in the whole health service.”* (Professional, Focus Group 3)

Although the situation had improved slightly since the easing of the pandemic, some professionals reported how delayed diagnosis continued to be an issue, particularly due to the pressures GP surgeries were experiencing, and the reduction of face-to-face appointments. This made the work of those in PEoLC more challenging, as they were unable to build relationships with patients and families at such a difficult and complex time and prior to having difficult conversations:

“*I'm walking into houses where people are completely oblivious of where they're at because they have not had as much face-to-face. We haven't been able to build those conversations; build the foundation.”* (Professional, Focus Group 4)

Some professionals did comment, however, that the increase in remote working since the pandemic could be viewed as a positive, particularly for those based in more remote locations:

“*Some of the things coming out of Covid have been good in terms of remote working.”* (Professional, Focus Group 7)

In addition to the reduction in face-to-face appointments, the impact of wearing masks and other personal protective equipment (PPE) was also discussed by participants. Masks were still being worn in some hospices and staff felt this made their work more challenging:

“*I would say there's still that challenge of coming out of Covid, especially here. Like, we're still in masks and things so it's quite difficult. Down the ward and in clinical area we're still in masks and that comes with its own challenges coming out of Covid. I know it's obviously got a lot easier as restrictions have lifted but it's still quite a challenge for us, I would say.”* (Professional, Focus Group 2)

Similarly, some patients recalled how difficult it had been to have conversations while wearing masks and how it had taken away the personal element of interactions:

“*That was the worst thing about Covid, wasn't it, the masks. With conversing, you know. Contact, physical contact with people, you haven't got it. And [it's] so, so necessary I think, when you're talking, to be able to see the full face when you're talking. So wrong.”* (Family carer, Focus Group 13)

As well as the practical issues highlighted above, participants discussed the emotional impact that the pandemic had on both staff and patients and families. Some professionals described how emotionally draining the pandemic had been for those working through it and the subsequent challenges this presented in retaining staff:

“*I think probably, you know, a lot of people in the health professions are very tired. COVID took an awful big whack out of people. It's amazing that people keep going, so I think there's a whole area for kind of looking at […] how they keep going. Where the battery and their energy comes from. Or not. And as we know […] kind of leaving health professions and vacancies and then the resources impact and then that's just, you know, an increasing burden for everyone.”* (Professional, Focus Group 7)

Professionals were also very aware of the emotional impact on patients and families. Participants observed how some people were traumatized by their experiences of hospitals and particularly the levels of death and dying during the pandemic. They felt this would affect how they coped with death in the future:

“*In palliative care we've always talked […] about a good death and good communication, you know. Good bereavement support enables people to deal healthily with the next death in their life… I think there's going to be a massive wave – in my humble opinion – of people that are really not going to be able to cope with bereavement, death and dying, another hospital admission, because they've had such horrific experiences during Covid.”* (Professional, Focus Group 4)

Linked to this, some professionals felt that negative experiences of health and care service provision during the pandemic had resulted in patients and families lacking trust in the system, leading them to question the care they received:

“*I would definitely think a lot of people – from our side of it – have definitely lost trust in the system. I mean, you see families coming in to see their loved ones and they're asking so many more questions, regarding those like issues, than pre-Covid. And you can – as a healthcare, you can definitely see the impact it's had. They're a lot more anxious than what they used to be. It's like, I think they just worry that the care's not going to be provided anymore.”* (Professional, Focus Group 4)

### A reluctance to have conversations about death and dying

The COVID-19 pandemic media coverage meant that the topic of death and dying became a topic of conversation across many households (Pentaris, 2022). Many of the professionals working across PEoLC pathways felt this was an opportunity for them and the wider public to talk more freely about death and dying:

“*…the world talked about death and dying, universally in a way we never have before. And conversations were had in every road on every street in every newspaper about dying…you know, we're perhaps in a place in time which we've never been at before where everybody's acutely aware and talking about the fact that we do all die. And it could happen sooner than you think...”* (Professional, Focus Group 3)

However, some participants felt that this newfound opportunity to talk about death and dying remained a cultural challenge. They suggested that conversations on these topics remained a taboo in the UK, as shared by these bereaved family members:

“*I think we need awareness. Everybody dies at some time…we've come away from – the Victorians were great on it. They, they talked about death all the time and it was something very important to them and they celebrated it in, in a way or they recognised it. Whereas now, it's like “Ooh”.”* (Bereaved Wife)

“*Yeah, but death in this country I think is a taboo subject.”* (Bereaved Husband)

“*Yeah, taboo subject.”* (Bereaved Wife) (Focus Group 11)

Professionals believed that the topic of death and dying had not yet been normalized, and until it was, these would remain challenging conversations:

“*…we don't treat death as a normal entity... You know, we have a birth plan, but we don't have a death plan. So, the reality… Isn't it? Dare I throw? You know, be the Devil's advocate. Isn't it about time we started to normalise it?”* (Professional, Focus Group 4)

They suggested that the delay in talking about death and dying was due to a model of healthcare that primarily focuses on cure and treatment:

“*I think it's a lack of wanting to talk about death. We're always promoting curative treatment and, “This is what we're going to do.” Consultants will say, “Yes, have this next round of chemotherapy”…we will know it's – that – and the person comes back from that treatment and the next day…dies.”* (Professional, Focus Group 4)

However, many acknowledged that talking about death and dying to patients and families was difficult for professionals, especially those with less experience, training and skills in that area:

“*I think there's still an issue with communication around death and having open and honest discussions, people don't use the word dying…And that is a problem with more junior staff or, you know, less experienced staff. They haven't got those skills to have that really honest conversation.”* (Professional, Focus Group 7)

There was also the concern amongst professionals that there was a particular gap in PEoLC conversations happening between the treatment and palliative care stages:

“*…the interface between oncology and palliative care…there's that common thing that patients have said to me about…there's nothing more to be done, or there's nothing more, or they feel kind of abandoned. Rather than perhaps having that conversation around we can't do any active treatment for you at the moment…you know, pass on or talk with our colleagues who can support you in the next phase of this illness.”* (Professional, Focus Group 7)

To improve the quality of these conversations, professionals suggested that Advance Care Planning (ACP) could be something people could begin to formulate even before they became unwell and nearing the end of their life. However, it was agreed that this required a better public understanding of the benefits of such conversations:

“*It's planning for end of life before you get there. It's all about the advanced care planning really, isn't it, and a better understanding of those sort of decisions that you suddenly have to get asked to make when you're really ill… If, if we could just have that better public understanding then it would – it would just make everyone's job a lot easier.”* (Professional, Focus Group 5)

It was suggested that if ACPs were better understood and used, people would perhaps be more prepared for their own deaths:

“*Early conversations…having those conversations really early so nothing is a surprise, so that everything is in place, you know DNR (Do Not Resuscitate), where they want to die, who, what, how they want to die. What they want to do before they before they die and how we can support them, to die, to have a good death.”* (Professional, Focus Group 7)

This professional shared their personal experience of how not being prepared for the death of a parent had impacted on them and their family:

“*…my dad died really suddenly so we didn't have any of those discussions or things…you want to get everything right and you do go into panic mode. Like, “What would he have wanted?” We didn't discuss anything…I think it would have been nice if we'd have had those conversations in advance and gone, “Oh, this is what he would have wanted to do and have” …”* (Professional, Focus Group 3)

Nevertheless, it was agreed that not everybody is ready to talk about death and dying openly and these conversations needed to be personalized and happen when the person was ready:

“*Somebody said to me, “It's like knocking on that door tentatively and seeing how the land lies before actually whether that door will open for you, or it will slam”.”* (Professional, Focus Group 4)

## Discussion

This study has brought together patients, families, and professionals in focus groups to explore their experiences of care. Five themes (a lack of palliative and end of life care resources; poor communication, collaboration and co-ordination across providers; inequalities in the access and provision of care; the impact of the COVID-19 pandemic; and a reluctance to have conversations about death and dying) have been presented in this paper. Although the COVID-19 pandemic was found to have had some impact on the participants' experiences of care, many of the barriers to care they faced predated the pandemic, and many factors continue to present.

The current lack of resources and funding in PEoLC was identified as an area of concern for many participants. Professionals shared the difficulties faced in identifying, recruiting and retaining staff with the appropriate skills in specialist care across the community, social care, and in acute hospitals. This is particularly challenging as the demand for palliative care is increasing as people live longer and present with multiple complex conditions (UK Parliament, 2022). It has been widely documented in recent research that services are struggling to manage this increased demand due to a lack of specialist PEoLC provision in hospitals and across the community (Mason et al., 2022; UK Parliament, 2022).

The lack of home-based and out-of-hours care for people at the end of their lives was described as particularly challenging in the study, with some services still operating on a Monday–Friday, 9 a.m.−5 p.m. basis. Patients and families shared their accounts of out-of-hours visits to emergency departments, and accessing out-of-hours medication was reported to be particularly difficult, especially in rural areas. Previous research has identified the challenge to timely access of care (Best et al., 2015) highlighting the value of designated phone lines for out-of-hours care (Pask et al., 2022), and the need for clearer information on how to access out-of-hours support (Mason et al., 2022).

Inequalities in access to care, across different groups, in different locations, was discussed by many participants. The existence of these inequalities in provision is well documented in recent literature (Baylis et al., 2023; National Palliative and End of Life Care Partnership, 2021). Participants' accounts described a “postcode lottery” effect, whereby geographical location influenced the availability and quality of the care and support available. This aligns with prior research which has indicated that people living in rural areas often have more difficulty accessing PEoLC services than those who live in urban or suburban areas (Chukwusa et al., 2019). Mackett (2014) reported that poor transport links can significantly hinder access to health and care provision in rural areas. This sentiment was echoed by the study participants, who described the substantial distances some patients travelled to receive care. This issue was compounded by the scarcity of reliable and affordable transport options, making it difficult for patients to attend appointments. Additionally, longer travel times for healthcare professionals can lead to delays in symptom management, causing distress for patients and their families.

Beyond geographical disparities, professionals discussed the challenges of providing inclusive and accessible care to all groups, particularly disadvantaged and seldom heard groups. A recent systematic review highlighted that persistent inequalities in hospice care provision exist, as “patients without cancer, the oldest old, ethnic minorities and those living in rural or deprived areas are under-represented in hospice populations” (Tobin et al., 2022, p. 142). Despite hospices' efforts to improve inclusivity, participants remained concerned that some patient groups were still not able to access specialist PEoLC. This disparity points to a broader systemic issue within health and care provision, where resource allocation and service availability are inconsistent (see also Cai and Lalani, 2022).

The study suggested that there was a challenge for people who did not have a single professional co-ordinating their care. This was especially problematic for those who decided to be cared for at home, where many services could be involved. The lack of co-ordination from care providers meant that family carers often had to take on that role (Reeves et al., 2020; Standing et al., 2020) resulting in them feeling overburdened and struggling to cope (Remawi et al., 2023). Fragmentated and disjointed care provision was not only distressing and frustrating to patients (Whitehead et al., 2022) and family carers (Harrison et al., 2022), but also the professionals. These findings are echoed by those of Whitehead et al. (2022) who also report that co-ordination challenges can lead to omissions or errors in information sharing due to poor communication. Participants also highlighted a lack of bereavement support options. Bereavement support is effective in reducing grief, depression and anxiety (Kustanti et al., 2021). However, this needs to be made available to people at the point they feel ready for it and in a format that is accessible to all.

Services and systems are often isolated from one another across PEoLC networks. This means that access to information can be challenging due to the variety of patient record systems in operation. However, patients and their family carers are often unaware of this, trusting that information is shared and communicated across a single and united health and care system (Standing et al., 2020). The study found that this lack of co-ordination and continuity meant that mixed messages are commonplace, particularly around the patient's prognosis or treatment. This was particularly evident when patients transitioned between active treatment and PEoLC pathways (Whitehead et al., 2022). A collaborative approach to PEoLC will continue to support disease treatment and control, but will also give equal status and time to PEoLC discussions and decision-making (Hugar et al., 2021). Murray and Amblàs (2021) suggest that the early and gradual introduction of palliative care could improve overall end of life care and reduce patient suffering.

The study found that palliative and end of life conversations are important, however, the timing of these conversations is crucial. It was acknowledged that instigating these conversations can be complicated due to the uncertainty of illness and disease, the unpredictability of a patient's response to treatment and knowing when the patient and family are ready to have that conversation (Remawi et al., 2023). However, some of the participants believed that the delay in having these conversations was because some health professionals were primarily focused on cure, and therefore did not consider the alternative of the patient dying. This can lead to professionals being unprepared or reluctant to discuss PEoLC in an open sensitive manner (Selman et al., 2017; NICE, 2019). The medicalization of death (Murray and Amblàs, 2021) and curative cultures (Selman et al., 2017) have both been cited as factors contributing to the delay in PEoLC conversations taking place. Having a medical focus on cure “at all costs” means that healthcare professionals are often prevented from offering the pastoral approach to care they may wish to provide (Van Brummen and Griffiths, 2013).

One way in which to support PEoLC conversations, referenced by participants, was the discussing, formulating and sharing of Advance Care Plans (ACP), also known as a future care plan or anticipatory care plan (McMahan et al., 2024). These care plans allow the person to make decisions and have choice about their future care, treatment, and even place of death. They are an opportunity to share “what matters to them” (Marie Curie, 2024). McFarlane et al. (2024) found that ACP activity did increase during the COVID-19 pandemic, when death and dying conversations were more likely to take place. However, some participants referred to the topic as a taboo. This is somewhat supported by a survey published by Marie Curie (Nelson et al., 2021) which found that 51% of the UK public thought we did not talk about death and dying enough, often as it was assumed a taboo topic. They also found that although the UK public reported to be comfortable about talking about death and dying, very few had done so (Nelson et al., 2021).

This study suggested the COVID-19 pandemic also affected PEoLC in other ways. The emotional impact of the pandemic on both health and care professionals, and patients and families were described as profound. Similar to other studies, many professionals experienced subsequent burnout and fatigue (Willis et al., 2023) and the intense workload and emotional strain have contributed to higher turnover rates and challenges in staff retention (Shanafelt et al., 2020). This has ongoing implications for workforce sustainability in health and care, and in PEoLC more specifically, which relies heavily on experienced and emotionally resilient professionals. Professionals were also aware that some patients and families were left traumatized by their healthcare experiences during the pandemic. This finding has been supported by others (Lourenço et al., 2024).

The implementation of safety protocols to control the spread of the virus impacted on relationships between professionals and patients and families (Lourenço et al., 2024). Participants highlighted the difficulties in conveying empathy and understanding through masks, which obscured facial expressions and hindered non-verbal communication. The reduction in face-to-face appointments and the replacement with phone and video consultations also appeared to pose a challenge in building trust and effective communication with patients and their families. This is particularly pertinent in PEoLC, where the quality of interpersonal relationships can significantly influence patient outcomes and family satisfaction (Kuosmanen et al., 2021). However, one of the most striking clinical impacts of the pandemic found in this study was the exacerbation of delayed diagnoses, resulting in patients being referred to palliative care at much later stages. This has also been reported by others (Lai et al., 2020; Maringe et al., 2020; Osei et al., 2023). Despite the unprecedented challenges presented by the pandemic and the shift to remote working, although problematic for some, it was also reported to be beneficial by health and care professionals working in remote locations. Diversifying the delivery of care can improve access to specialist PEoLC services, particularly in under-served areas to enhance care equity (Calton et al., 2020).

## Strengths and limitations

This study brings together the experiences of people living at the end of life and their family carers, people who are bereaved, as well as professionals from different health and care settings. This supports the triangulation of experiences and perspectives. The study participants were recruited by six hospices situated across South-West England through their extensive networks across health and care, and patient and family groups. Although this paper provides a regional picture of the barriers to PEoLC experienced by professionals, patients and families, the insights revealed apply beyond South-West England. Inequity of access and discomfort around dying, for example, are two barriers that are faced not only nationally, but globally, particularly in lower to middle income countries (Peeler et al., 2024).

It is acknowledged that the study focus was the barriers and challenges to care and therefore the positive aspects of PEoLC provision, of which there are many, are not captured or shared in this paper. Furthermore, some selection bias may be present due to some of the participants receiving specialist PEoLC/support from the recruiting hospices. It must also be acknowledged that although the sample is regionally representative, the region studied is not demographically diverse, particularly in terms of ethnicity.

We recommend that future research on this topic should have a wider recruitment scope, e.g., with a wider range of participants with diverse experiences of care from different ethnic and socio-economic backgrounds, and particularly with representation from disadvantaged and seldom heard groups. Another area of care that requires considerable thought and additional research is the exploration of geographical inequalities such as rurality and the provision of out-of-hours PEoLC, specifically in terms of symptom control, pain management, and emotional support.

This focus group study provides a platform for further research, particularly research that captures experiences and perceptions across a wide range of care providers and patient groups. This will require research methods that are innovative, inclusive and accessible to all groups of patients, and not just the “easy to reach.”

## Conclusion

This study brings together the voices of patients, family and professionals from different care settings across a geographical area of the UK. Understanding the experiences and the perceived barriers to care of all those involved in PEoLC is key to being able to develop and transform care. This study builds on previous research and highlights the importance of investment, planning, practice change and policy development across PEoLC to address inequality and barriers to care. There is an urgent need for wider systems-level and participatory research that involves people near the end of life, alongside those who care for them. Ultimately, there is a necessity for a collaborative and co-ordinated approach across both practice and research, addressing what is important to those providing, and most importantly, those receiving care at the end of their lives.

## Data Availability

The original contributions presented in the study are included in the article/supplementary material, further inquiries can be directed to the corresponding author.
